# Runt-related Transcription Factors and Gene Regulatory Mechanisms in Skeletal Development and Diseases

**DOI:** 10.1007/s11914-023-00808-4

**Published:** 2023-07-12

**Authors:** Hironori Hojo, Shinsuke Ohba

**Affiliations:** 1 Division of Clinical Biotechnology, Center for Disease Biology and Integrative Medicine, Graduate School of Medicine, The University of Tokyo, 7-3-1 Hongo, Bunkyo-Ku, Tokyo, 113-8655 Japan; 2 Department of Bioengineering, Graduate School of Engineering, The University of Tokyo, Tokyo, 113-8655 Japan; 3 Department of Tissue and Developmental Biology, Graduate School of Dentistry, Osaka University, 1-8 Yamadaoka, Suita, Osaka 565-0871 Japan

**Keywords:** RUNX, Gene regulatory mechanisms, ChIP-seq, Skeletal development, Skeletal diseases

## Abstract

**Purpose of Review:**

Runt-related transcription factors (RUNX) play critical roles in skeletal development, metabolism, and diseases. In mammals, three RUNX members, namely RUNX1, RUNX2, and RUNX3, play distinct and redundant roles, although RUNX2 is a dominant factor in skeletal development and several skeletal diseases. This review is to provide an overview of the current understanding of RUNX-mediated transcriptional regulation in different skeletal cell types.

**Recent Findings:**

Advances in chromatin immunoprecipitation and next-generation sequencing (ChIP-seq) have revealed genome-wide RUNX-mediated gene regulatory mechanisms, including their association with cis-regulatory elements and putative target genes. Further studies with genome-wide analysis and biochemical assays have shed light on RUNX-mediated pioneering action and involvements of RUNX2 in lipid–lipid phase separation.

**Summary:**

Emerging multi-layered mechanisms of RUNX-mediated gene regulations help us better understanding of skeletal development and diseases, which also provides clues to think how genome-wide studies can help develop therapeutic strategies for skeletal diseases.

## Introduction

The runt-related transcription factor (RUNX) was first identified as an essential regulator of early embryonic segmentation in Drosophila [1]. RUNX genes are conserved in most metazoan genomes such as those of insects and vertebrates. In mammals, there are three RUNX transcription factors (TFs), namely RUNX1, RUNX2, and RUNX3 [2]. RUNX members are expressed in distinct cell types and play pivotal roles in multiple biological processes, such as skeletal development, metabolism, and diseases (see reviews [2–4] for details). Briefly, *Runx2* is essential for osteoblast specification and chondrocyte hypertrophy. During osteogenesis, skeletal progenitors initially commit to forming RUNX2-positive osteoblast precursors, which then differentiate into RUNX2- and SP7-double-positive osteoblast precursors [5]. Sp7 is another master regulator of osteoblast specification; *Sp7* is genetically downstream of *Runx2* [6]. In *Runx2*-deficient mice, osteoblast differentiation was arrested and no *Sp7* was detected [6]. In chondrogenesis, *Runx2* is weakly expressed in proliferating columnar chondrocytes but is markedly upregulated as chondrocytes exit the cell cycle and become pre-hypertrophic and subsequently hypertrophic chondrocytes. The ectopic expression of *Runx2* in columnar chondrocytes accelerates chondrocyte hypertrophy [7–9] and the knockout of *Runx2* prevents normal hypertrophic cartilage mineralization [10].

*Runx1* and *Runx3* expression partially overlaps with *Runx2* expression; these have redundant and distinct roles in association with RUNX2 in skeletal cells. In osteogenesis, RUNX1 and RUNX3 positively regulate osteoblast proliferation and differentiation, and RUNX1 functions as a compensatory factor for RUNX2 [11, 12]. During chondrogenesis, *Runx1* is expressed in mesenchymal cells and proliferating chondrocytes, whereas *Runx3* is highly expressed in hypertrophic chondrocytes. The complete knockout of both *Runx1* and *Runx2* results in sternal abnormalities [13], whereas the double knockout of *Runx2* and *Runx3* results in a complete loss of cartilage and bone mineralization [10].

The crucial roles of *Runx2* and redundant or distinct roles of *Runx1* and *Runx3* are also highlighted in skeletal metabolisms and diseases in adult stages. Bone mineral density decreases upon *Runx2* deficiency in adult stages [12], whereas compared with *Runx2*-deficiency in adult skeletons in mice, *Runx1*- or *Runx3*-deficiency results in milder phenotypes [11, 14]. RUNX1 and RUNX2 are also involved in the formation of a niche and the maintenance of hematopoietic stem cells in bone marrows [15]. During osteoarthritis (OA) progression, RUNX1 and RUNX3 mainly exhibit protective functions, whereas RUNX2 exhibits both anabolic and catabolic functions [16–19]. In this review, we provide an overview of the current understanding of gene regulatory mechanisms and emerging RUNX-mediated transcriptional regulation in skeletal cell types and their states. We then discuss future perspectives on how to apply knowledge from genome-wide studies to the development of therapeutic strategies for skeletal diseases.

### Gene Regulatory Mechanisms

**Fig. 1:**
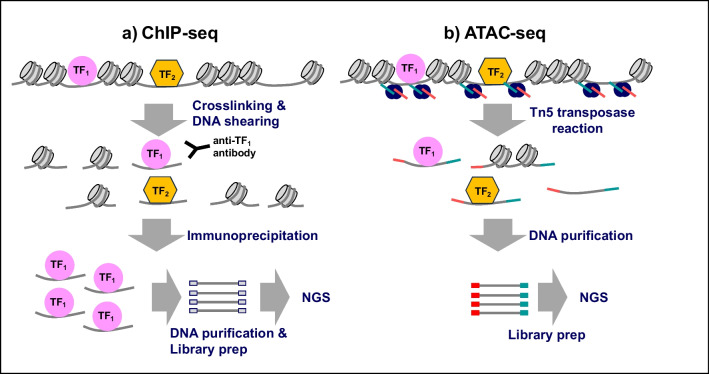
Overview of ChIP-seq and ATAC-seq experiments. **a** In ChIP-seq experiments, chromatin DNA is first cross-linked with formaldehyde, followed by DNA shearing using either sonicator or enzymes to obtain DNA fragments of 100 to 600 bp. Then, the DNA-protein complex is immunoprecipitated by the specific antibody for a protein of interest. After protein digestions, purified DNA fragments are amplified for NGS analysis. The output data are sequences of regions that were interacted with the protein of interest. **b** In ATAC-seq, chromatin DNA is directly reacted with Tn5 transposase which associates with open chromatin regions. Tn5 transposase then cut open chromatin regions with tagging specific nucleotide sequences which are used for DNA amplification for NGS. The output data are sequences of Tn5 accessible regions, i.e., open chromatin regions, in the genome

Transcription is a key process where genes are transcribed into proteins that exhibit their biological functions. Transcript profiles are distinct depending on the cell type and physiological and pathological conditions. Transcription is tightly regulated by enhancers, cis-regulatory elements (CREs), where multiple transcriptional regulators coordinately act [20, 21]. To understand gene regulatory mechanisms at the genomic level, chromatin immunoprecipitation and next-generation sequencing (ChIP-seq) and related assays have been developed over the past two decades [22]. ChIP-seq for a TF provides TF–DNA-binding profiles and the mode of action of TFs on the genome (Fig. 1a). ChIP-seq for histone modifications provides a broad range of epigenetic perspectives. For example, trimethylation at the 4^th^ lysine residue of the histone H3 protein (H3K4me3) represents an active promoter, and the acetylation of the lysine residue at the N-terminal position 27 of the histone H3 protein (H3K27ac) indicates an active enhancer. Overall, these provide a theory called “histone code”: the combination of histone modifications defines an epigenetic status in cells [23]. In addition, an assay for transposase-accessible chromatin using sequencing (ATAC-seq) shows “chromatin accessible” regions for transcriptional regulator binding [24] (Fig. 1b). Chromatin accessibility is strongly associated with cis-regulatory actions [25]. Given the requirement of a small number of cells, this technique has been widely used with a limited number of cells, such as sorted cells.

### RUNX ChIP-Seq Analysis in Osteogenesis

ChIP-seq studies on RUNX2 and histone modifications during osteogenesis were first reported by three independent groups in 2014. Several studies have provided insights into RUNX2-mediated gene regulation at the genomic level in osteoblasts. First, these studies identified putative CREs in osteoblasts [26–28]. CREs are located not only in the flanking regions of transcription start sites (TSSs) but also > 500 bp away from the TSS. These results indicate that RUNX2–DNA interactions occur at distal enhancer and proximal promoter regions [28, 29]. Meyer et al. further highlighted the importance of distal enhancers by analyzing *Mmp13* CREs. The distal *Mmp13* enhancer was strongly responsive to RUNX2 expression according to the reporter assay, *Mmp13* expression was markedly suppressed by the deletion of the distal enhancer using CRISPR/Cas9 technology in vitro [29].

The examination of RUNX2-binding sites at various stages of osteoblast differentiation revealed dynamic changes in the RUNX2-DNA-binding sites. Wu et al. performed a clustering analysis of RUNX2 binding in conjunction with transcript profiles at various time points in an in vitro differentiation culture of the pre-osteoblastic MC3T3-E1 cell line [27]. The results showed that a subset of RUNX2 ChIP-seq peaks was highly associated with osteoblast-related genes that were activated upon osteoblast induction [27]. Another cluster of RUNX2 peaks, which was lost upon osteoblast induction, was related to biological functions in other cell lineages, including fat cell differentiation, leukocyte migration, and erythrocyte differentiation [27]. These findings suggest that RUNX2 may have broader interactions prior to osteogenesis or that binding to non-osteoblast targets may suppress non-osteogenic pathways of cell commitment [27]. A recent RUNX2 ChIP-seq study supported this negative action; RUNX2 and early growth response protein 1 cooperatively inhibited the expression of HtrA serine peptidase 1 (*Htra1*) by binding to its distal enhancers [30].

### RUNX ChIP-Seq Analysis in Chondrogenesis

The ChIP-seq of RUNX1, RUNX2, and RUNX3 has been performed in chondrocytes in vitro [16, 19]. Although, as described earlier, the roles of RUNX members differed in anabolic and catabolic functions in OA models [16–19], overall motif enrichment and the proportion of the ChIP-seq peak distance from the TSS were similar among the RUNX members. The RUNX consensus motif was highly enriched and more than half of the DNA-binding regions were far from the TSS. However, the putative RUNX target genes differed among the RUNX members. Zhou et al. performed RUNX1 ChIP-seq using chondrocytes. The assay revealed that RUNX1 was highly associated with genes related to the Hippo signaling pathway and skeletal system development [19]. They identified RUNX1 target genes, including transmembrane anterior posterior transformation 1 (*TAPT1*), protein RIC1 homolog (*RIC1*), and fibroblast growth factor 20 (*FGF20*). The expression of these genes was downregulated in an osteoarthritic mouse model, whereas their expression was rescued by RUNX1 overexpression, in conjunction with protection against cartilage destruction [19].

Nagata et al. performed RUNX3 ChIP-seq on primary cells isolated from the superficial zone of the mouse articular cartilage [16•]. The assay showed that RUNX3 was highly associated with genes related to extracellular structure organization and collagen fibril organization. Further analysis using reporter assays and histological analysis in *Runx3*-deficient mice showed that RUNX3 directly regulated aggrecan (*Acan*) and proteoglycan 4 (*Prg4*). *Prg4* functions as a boundary lubricant in the articular cartilage to decrease wear and friction. *Acan* is another anabolic factor in the cartilage. Thus, RUNX3 likely exhibits anabolic functions by regulating *Prg4* and *Acan* during cartilage metabolism.

 Nagata et al. performed RUNX2 ChIP-seq using primary chondrocytes in vitro [16•]. Here, the cells were treated with interleukin (IL)-1β to establish a model of inflammation. The analysis showed that RUNX2 was highly associated with genes related to collagen fibril organization in the cells regardless of IL-1β treatments. Further gene expression analyses in *Runx2*-deficient chondrocytes treated with IL-1β revealed that the expression of *Col2a1*, a cartilage anabolic factor, was downregulated in *Runx2*-deficient cells treated with IL-1β, whereas it did not significantly change between the control and *Runx2*-deficient cells without IL-1β treatment. Motif analysis and reporter assays further showed that SRY-box transcription factor 9 (SOX9), a chondrocyte master regulator, compensated for RUNX2 during *Col2a1* transcription. However, upon inflammation, SOX9 expression decreased, resulting in a decrease in anabolic factors including *Col2a1*. On the other hand, *Mmp13*, a catabolic factor, was downregulated because of *Runx2* deficiency regardless of IL-1β treatment. These data suggest that RUNX2 acts as an anabolic and catabolic factor in different conditions, where Sox9 possibly compensates for RUNX2-mediated regulatory networks during the progression of OA. Notably, in the RUNX1 ChIP-seq analysis, the authors focused on RUNX1–DNA binding from the < 3 kb proximal promoter, whereas in RUNX2 and RUNX3 ChIP-seq studies, the authors investigated DNA binding from the > 500 bp distal enhancer far from the TSS. Thus, a comprehensive analysis in the same setting will help better understand detailed gene regulatory mechanisms underlying the different biological outcomes of the effects of the three RUNX members.

### Comparative Analysis of RUNX2 ChIP-Seq in Osteoblasts and Chondrocytes (Fig. 2a)

**Fig. 2:**
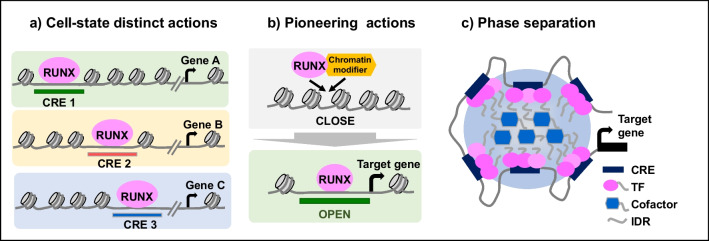
Emerging RUNX-mediated gene regulatory mechanisms. a Different CREs are used for RUNX binding in a cell-type- or cell-state-dependent manner. The binding regions were also not the same among RUNX factors. These variations may explain the distinct biological outcomes. b RUNX factors are likely to have pioneering functions. RUNX binds to closed chromatin regions and opens the chromatin for later activation. Chromatin modifiers physically interact with RUNX and are likely involved in its pioneering action. c RUNX is involved in liquid–liquid phase separation. Interactions between the IDRs in the N-terminus of RUNX2 and cofactors are crucial for its formation. However, whether RUNX1 and RUNX3 exert similar effects on gene regulation remains unclear. RUNX, runt-related transcription factors; CRE, cis-regulatory element; TF, transcription factor; IDR, intrinsically disordered regions

We recently reported a RUNX2 ChIP-seq study on primary osteoblasts and chondrocytes coupled with the ATAC-seq analysis of sorted skeletal cells in neonatal mice, providing insights into RUNX2-mediated regulatory mechanisms in a near in vivo setting [31••]. Because RUNX2 is essential for both osteoblast specification and chondrocyte hypertrophy, we aimed to understand cell-type-specific mechanisms. First, RUNX2 expression was highly associated with distinct CREs in osteoblasts and chondrocytes. For example, RUNX2 binding to the osteoblast-specific CRE was detected in the *Col1a2*-flanking region in osteoblasts but not in chondrocytes. RUNX2 binding to CREs in the *Col10a1*-flanking region was detected in chondrocytes but not in osteoblasts. Consistent with these results, chromatin accessibility showed distinct cell-type signatures. Overall, cell-type-distinct RUNX2-DNA binding highlighted distinct cell-type target genes. Osteoblast-specific RUNX2 targets included *Col1a1*, *Col1a2*, *Ostn*, *Fgf18*, *Dlx5*, and *Mef2c*, whereas chondrocyte-specific targets included *Col2a1*, *Col10a1*, *Bmpr1b*, *Sox5*, and *Creb3l2*. Common target genes are also present in osteoblasts and chondrocytes. Notably, even for common target genes, such as *Spp1*, the distribution of RUNX2–DNA binding and chromatin accessibility around the genes was different between osteoblasts and chondrocytes [32]. These results suggest that RUNX2-mediated CRE activities are more diverse and cell-type-specific than the outcome of RUNX2 association, that is, gene expression.

RUNX2 and cell-type-specific TFs are associated with CREs in osteoblasts and chondrocytes. FOX motifs were exclusively enriched in RUNX2-bound regions in hypertrophic chondrocytes. Gene expression analysis showed the high expression of related TFs, including *Foxa2*, *Foxa3*, and *Foxc1*, supporting their indispensable roles in hypertrophic chondrocytes [33–36]. In the case of osteoblast regions, the SP7–DLX motif [37] was specifically enriched in osteoblasts. *Sp7*, *Dlx3*, *Dlx5*, and *Dlx6* were highly expressed in the cells, supporting essential roles in osteogenesis [6, 38]. In addition to specific TFs, activator protein 1 (AP-1) and activating transcription factor (ATF) motifs were enriched in both cell types. This suggests that AP-1 and ATF are required in both cell types [39–43]. Because this type of assay relies on the DNA-binding actions of TFs, DNA-binding-independent actions, such as protein–protein interactions and post-translational modifications, are not detected. Thus, further proteomic analysis will help identify the entire complex of the transcriptional machinery.

### Pioneering Actions of RUNX Members Coordinating Chromatin Accessibility (Fig. 2b)

Recently, a model of “pioneer factors” has been proposed; “pioneer factors” are supposed to facilitate the opening of closed chromatin sites [44]. In this model, they bind to inaccessible genomic regions and recruit other TFs, cofactors, and chromatin modifiers to make the chromatin “accessible” to initiate transcription. Several lines of evidence support the notion that RUNX2 plays a pioneering role in osteoblast specification [31••]. First, the exogenous expression of *Runx2* in fibroblasts led to its DNA binding in closed chromatin regions, where chromatin accessibility was gained later [31••]. The high enrichment of the consensus RUNX motif in the altered genomic regions supports the idea that RUNX2 is directly associated with previously closed genomic sites. Second, a genetic study on mice revealed abundant changes in chromatin accessibility due to *Runx2* ablation in *Sp7*-positive osteoblast precursors [31••]. The “*Runx2*-dependent” regions were distal from the TSS, enriched with the consensus RUNX motif, and highly associated with genes related to skeletal system development [31••]. These results suggest that RUNX2 is required for chromatin accessibility in osteoblasts, which may underlie RUNX2-mediated regulatory mechanisms in osteoblast specification. A recent study showed that the ten-eleven translocation (TET) family of dioxygenases physically interacted with RUNX2; TET-mediated demethylation increased the chromatin accessibility of target genes by RUNX2 and facilitated RUNX2-regulated transcription [45•]. Thus, a transcriptional complex comprising RUNX2 and chromatin modifiers may facilitate chromatin accessibility.

Consistent with RUNX2, RUNX1 and RUNX3 have been suggested to act as pioneer factors in different cell types [2, 46]. RUNX1 has been shown to shape the chromatin landscape in metanephric mesenchymal and hematopoietic cells [47, 48]. RUNX3 plays a pioneering role in cell-cycle progression. The runt domain of RUNX3 physically interacts with the bromodomain of BRD2, which interacts with the MLL1/MLL5 and SWI/SNF protein complexes to promote chromatin opening [49]. Further structural analysis of RUNX with chromatin modifiers will help understand how RUNX factors are associated with chromatin accessibility and how different RUNX factors act on the chromatin.

### Liquid-Liquid Phase Separation (Fig. 2c)

Phase separation is the basis for the formation of membrane-less organelles in cells and is involved in many biological processes. Recent studies have indicated that the assembly of transcription machinery at genomic sites occurs via liquid–liquid phase separation, leading to the formation of transcriptional condensates [50, 51]. At these sites, the clusters of enhancers are bound by master TFs with high densities of coactivators, forming super-enhancers [52]. Indeed, the intrinsically disordered regions (IDRs) in the N-terminus of RUNX2 have been reportedly involved in phase separation. Mutations in alanine repeat expansions in the RUNX2 IDR alter its phase separation capacity and transcriptional activity, which are involved in cleidocranial dysplasia [53••].

However, the involvement of other RUNX members in phase separation remains to be clarified. RUNX2 IDR sequences are not well-conserved compared with those of RUNX1 or RUNX3, suggesting that phase separation may occur only with RUNX2. However, a recent study showed that the RUNX2 repression domain at the C-terminus is required for phase separation in cardiomyocytes, where RUNX2 interacted with arachidonate 5-lipoxygenase [54]. This repression domain is partially conserved among the RUNX factors. Therefore, different RUNX domains may be required for phase separation with different partners.

## Conclusion

Genome-wide studies have provided valuable insights into diverse aspects of gene regulation, including DNA-binding regions, histone modification, chromatin accessibility, and lipid–lipid phase separation. RUNX plays a critical role in controlling gene regulatory networks via diverse mechanisms. The fields that still require further exploration are as follows: (1) the development of therapeutic strategies based on insights gained from genome-wide analyses and (2) a deeper understanding of the human genetic variants that contribute to diseases.

First, as described earlier, cis-regulatory actions are dynamic and cell-state-specific. Gene regulatory mechanisms underlying pathological conditions and tissue regeneration should be investigated. It is worth identifying disease-specific gene regulatory networks and manipulating them for treatment and disease diagnosis. CRISPR technology for epigenomic editing may be helpful [55]. Interestingly, a tissue repair–specific enhancer was identified in a fish study [56]; another study used this enhancer for gene delivery systems in mammals [57]. Similarly, skeletal disease–specific enhancers could be used as delivery systems for treatment in the future.

Second, from a human genetics perspective, extensive genome-wide association studies (GWAS) have identified associations between the human genome, skeletal development, and diseases [58, 59]. Notably, more than 90% of the genetic loci associated with diseases have been identified outside protein-coding regions, with enhancers accounting for approximately 40% of the non-coding regions [60]. Recent integrative analyses of GWAS have identified the effector genes of GWAS loci in osteoporosis and related skeletal diseases [58, 61, 62]. Thus, accumulating knowledge on gene regulatory mechanisms will provide a rich resource for connecting regulatory variants to human diseases. To bridge the gap between regulatory variants and diseases, we recently reported CRE profiling in human skeletal development using human pluripotent stem cells [63]. Although crucial CREs are conserved among species, human-specific single-nucleotide polymorphisms may also be involved in these diseases. Further studies on human CREs will help understand human variants underlying the molecular mechanisms of pathogenic conditions.
